# Crystal structures and Hirshfeld surface analyses of two precursors of the etoxazole metabolite ‘R8’

**DOI:** 10.1107/S2056989026004329

**Published:** 2026-04-29

**Authors:** Chaluvarangaiah Sowbhagya, Thaluru M. Mohan Kumar, Papegowda Bhavya, Holehundi J. Shankara Prasad, C. Harish Chinthal, Hemmige S. Yathirajan, Sean Parkin

**Affiliations:** a Department of Physical Sciences Amrita School of Engineering Amrita Vishwa Vidyapeetham, Bengaluru-560 035 India; b Department of Applied Sciences, New Horizon College of Engineering, Bengaluru-560 103, India; c Chemistry Department, Morarji Desai Residential Science PU College, Parshwaganahalli-513 101, India; d Chemistry Department, Vidyanidhi PU College, Tumkur-572 101, India; e Department of Studies in Chemistry University of Mysore, Manasagangotri Mysuru-570 006 India; f Department of Chemistry University of Kentucky,Lexington KY 40506-0055 USA; Institute of Chemistry, Chinese Academy of Sciences

**Keywords:** crystal structure, Hirshfeld surface, etoxazole metabolite R8, acaricide, insecticide

## Abstract

Etoxazole is an agricultural pesticide that degrades into potentially haza­rdous metabolites, necessitating a thorough understanding of their chemical properties for accurate environmental and health risk assessments. This study reports the synthesis and structural characterization of two precursors of an etoxazole metabolite designated ‘R8’: 1-(4-*tert*-butyl-2-eth­oxy­phen­yl)-2-hy­droxy­ethan-1-one, C_14_H_20_O_3_ (**I**), and 1-(4-*tert*-butyl-2-eth­oxy­phen­yl)ethan-1-one, C_14_H_20_O_2_ (**II**). Compound **I** crystallizes with the symmetry of space group *Pnma*, while **II** crystallizes as *P*2_1_/*m*. In both structures, the mol­ecules lie on crystallographic mirror planes (*Z*′ = 1/2) and exhibit a high degree of conformational similarity, differing primarily in the torsion angle of the *tert*-butyl group. The crystal packing in both structures is consolidated by weak C—H⋯π contacts that assemble the mol­ecules into columns parallel to the crystallographic *b* axes. Hirshfeld-surface analyses show that the inter­molecular inter­actions in both compounds are overwhelmingly dominated by contacts involving hydrogen atoms.

## Abstract 2

The crystal structures and Hirshfeld surface analyses of two precursors obtained in the synthesis of metabolite ‘R8’ of the insecticide/acaricide etoxazole are presented.

## 1. Chemical context

Etoxazole is a diphenyl oxazoline acaricide/insecticide, widely used to control mites and ticks on crops, fruits, vegetables, and ornamental plants (Wei *et al.*, 2014 ▸). It works by inhibiting chitin biosynthesis and causing adult pests to lay sterile eggs (Nauen *et al.*, 2006 ▸). In both environmental and biological systems, etoxazole degrades through hydrolysis, oxidation, photodegradation, and microbial transformation. In rat and human liver microsomes specifically, it undergoes enanti­oselective metabolism, with each enanti­omer degrading at a different rate (Yao *et al.*, 2016 ▸). These degradation processes form several metabolites that are not yet fully understood. Some may pose environmental and health risks, potentially exhibiting similar or higher toxicity than etoxazole itself (Sun *et al.*, 2019 ▸). Detailed study of these metabolites is therefore imperative for accurate risk assessment.

One significant metabolite has been designated ‘R8’ (FAO/WHO, 2011 ▸), systematic name 2-amino-2-(4-*tert*-butyl-2-eth­oxy­phen­yl)ethan-1-ol, C_14_H_23_NO_2_. Understanding the environmental behaviour, toxicity, and persistence of R8 and related compounds is critical for the remediation and risk assessment of etoxazole-related chemicals. The title compounds of this study, namely 1-(4-*tert*-butyl-2-eth­oxy­phen­yl)-2-hy­droxy­ethan-1-one (**I**) and 1-(4-*tert*-butyl-2-eth­oxy­phen­yl)ethan-1-one (**II**), C_14_H_20_O_3_ and C_14_H_20_O_2_ are precursors isolated during the synthesis of the R8 metabolite.
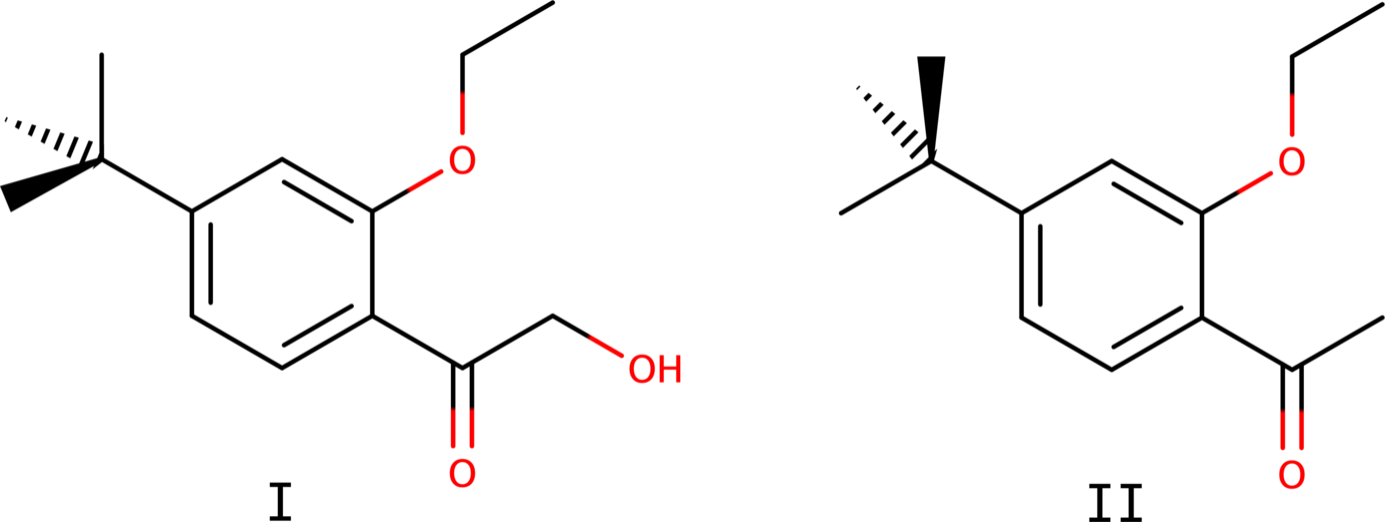


## 2. Structural commentary

**Figure 1:**
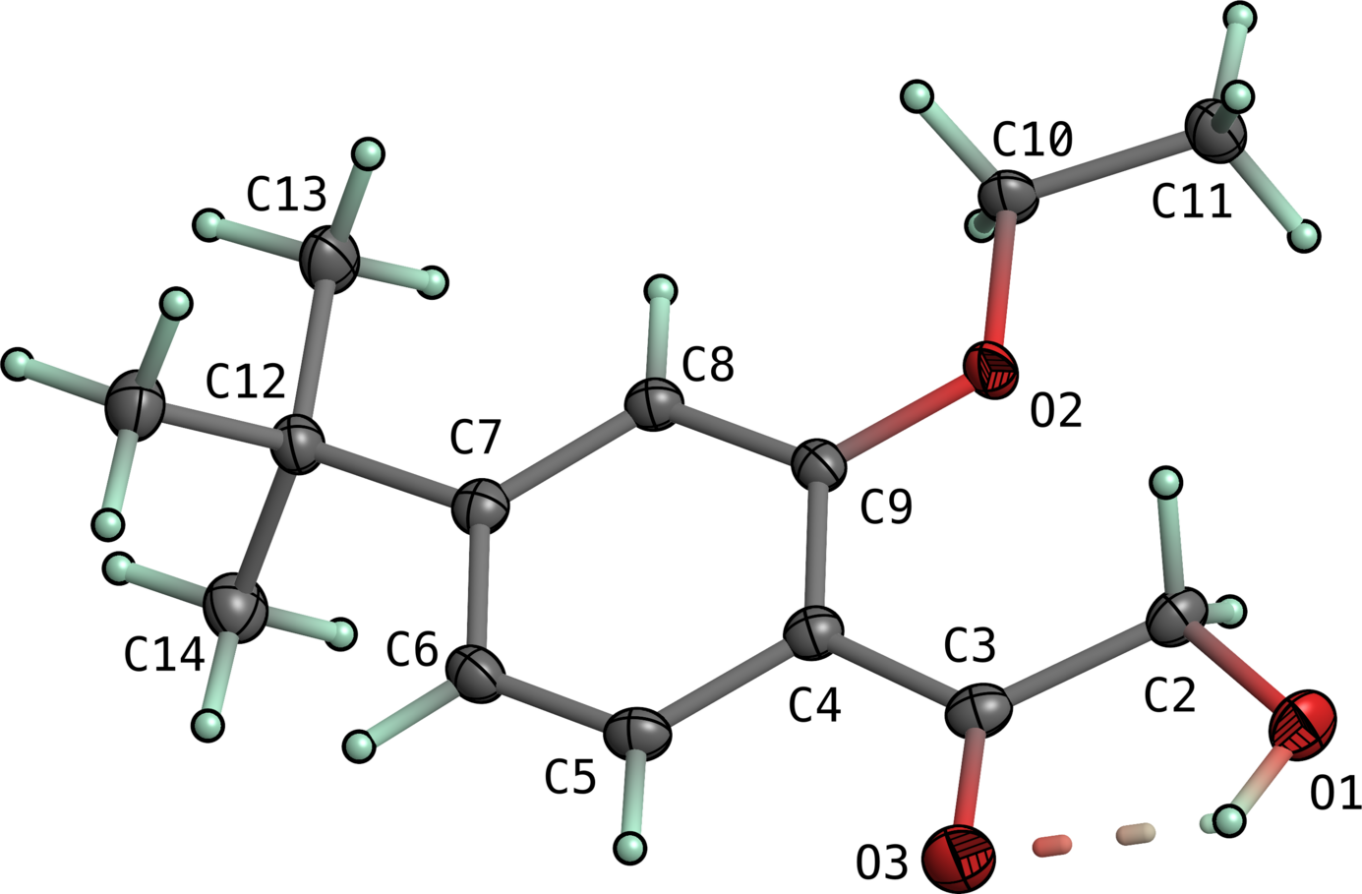
An ellipsoid plot of **I** (50% probability). Hydrogen atoms are drawn as small arbitrary spheres. An intra­molecular hydrogen bond is shown as a dashed line.

**Figure 3:**
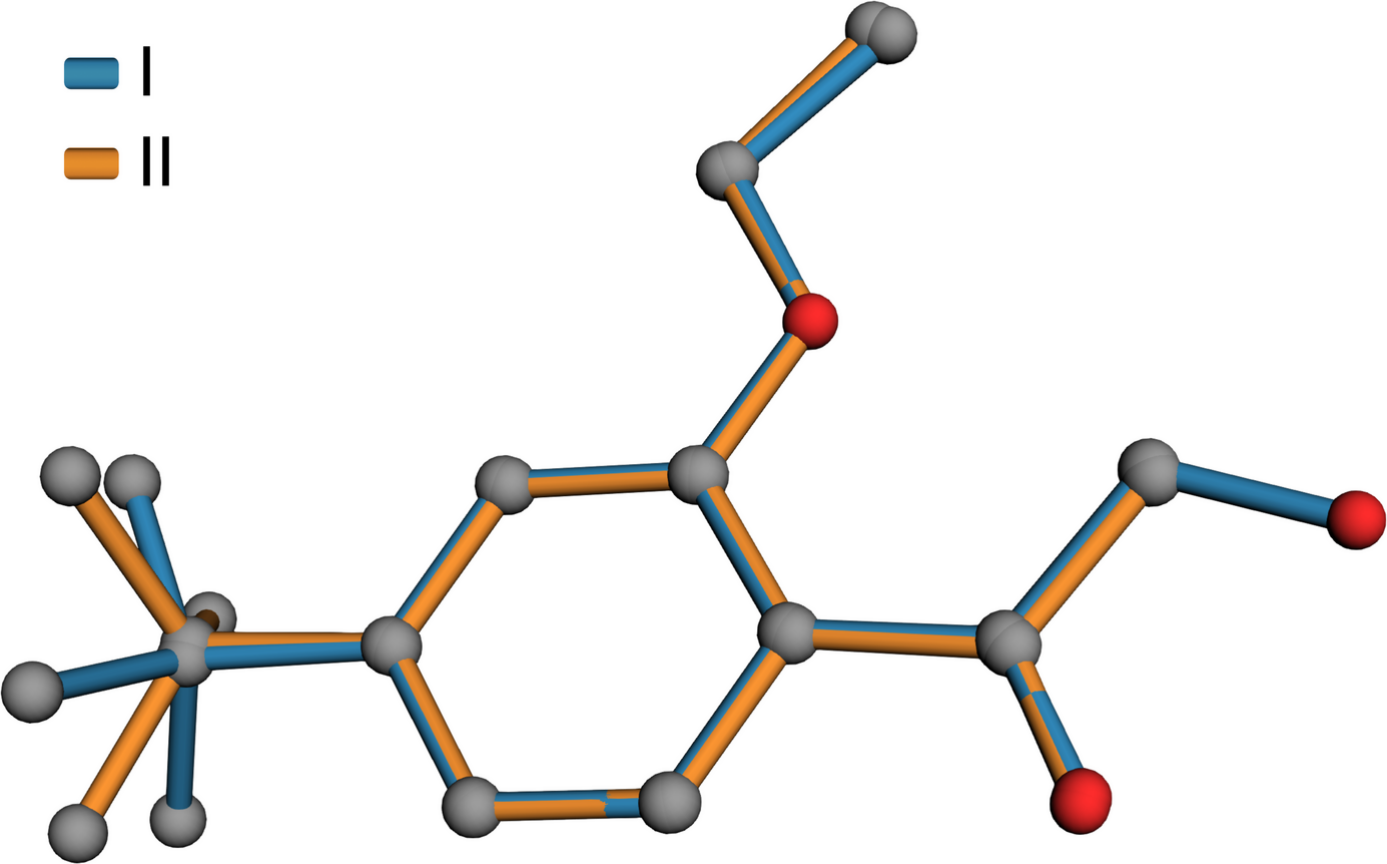
A least-squares-fit superposition of **I** (blue) and **II** (orange). The r.m.s. deviation of fitted atoms (*i.e.*, all atoms except the *tert*-butyl methyls is 0.0528 Å (grey spheres, left) and hydroxyl oxygen (red sphere, right).

The mol­ecular structures of both **I** and **II** consist of 4-*tert*-butyl-2-eth­oxy­phenyl moieties substituted at the 1-position by 2-hy­droxy­ethan-1-one in **I** and ethan-1-one in **II**. In both structures, the majority of atoms lie on crystallographic mirror planes. The mol­ecular structures are shown in Figs. 1 ▸ and 2 ▸. The space group of **I** is *Pnma*, while that of **II** is *P*2_1_/*m*, with both having *Z*′ = 0.5. There are no unusual bond lengths or angles in either structure. Given the similarity of the two mol­ecules and the fact that they each lie on crystallographic mirror planes, there is a remarkable degree of superpositional overlap, as evident from the least-squares overlay in Fig. 3 ▸. For the fitted atoms (O2, O3, C2–C12), the r.m.s. deviation is only 0.0528 Å. The most obvious conformational difference lies in the torsion of the *tert*-butyl group. In **I**, the C6—C7—C12—C13 torsion is 180°, while in **II** it is 0°, with both angles being constrained by their respective mirror planes. Structure **I** includes an intra­molecular hydrogen bond [O1—H1*O*⋯O3, *d*_*D*⋯*A*_ = 2.5518 (16) Å], which forms an *S*(5) ring motif (Etter *et al.*, 1990 ▸).

## 3. Supra­molecular features

There are no conventional inter­molecular hydrogen bonds in either **I** or **II**. The geometric criteria in *SHELXL* (Sheldrick, 2015*b* ▸) flag C11—H11A⋯O1^i^ [symmetry code: (i) *x*, *y*, *z* − 1] in **I** and C8—H8⋯O3^ii^ [symmetry code: (ii) *x* − 1, *y*, *z*] in **II** as ‘potential hydrogen bonds’, but the geometries involved (see Tables 1 ▸ and 2 ▸) suggest that these would be very weak.

**Figure 4:**
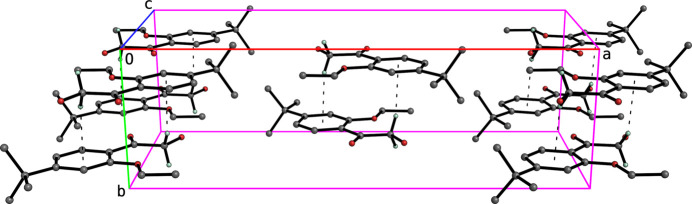
A perspective partial packing plot of **I** viewed slightly off the *c*-axis. C—H⋯π contacts are drawn as thin dashed lines, which connect the mol­ecules into columns that extend parallel to the *b*-axis.

In spite of the presence of benzene rings constrained to lie within planes parallel to *ac* in both **I** and **II**, neither structure has any π–π stacking. They do, however, each exhibit C—H⋯π contacts, as shown in Figs. 4 ▸ and 5 ▸, which lead to columns parallel to their respective *b* axes.

**Figure 6:**
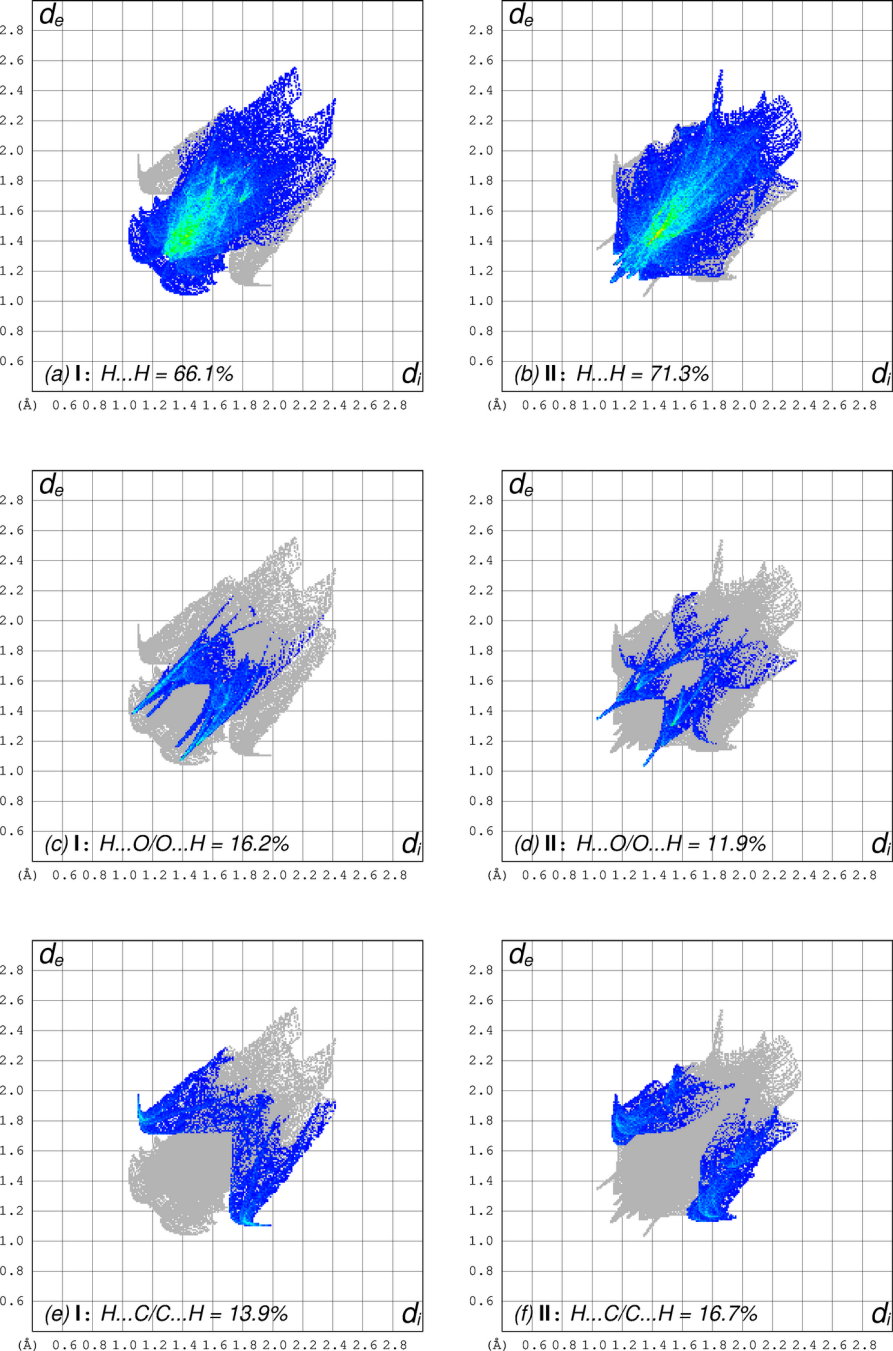
Hirshfeld-surface fingerprint plots of the most abundant types of atom-atom contacts in **I** and **II**. (*a*), (b) H⋯H contacts, (*c*), (*d*) H⋯O/O⋯H, (*e*), (*f*) H⋯C/C⋯H.

Hirshfeld surface analyses using *CrystalExplorer* (Spackman *et al.*, 2021 ▸) show that virtually all atom–atom contacts in both **I** and **II** involve hydrogen: 96.2% in **I**, 99.99% in **II**, with 66.1% and 71.3% being H⋯H contacts in **I** and **II**, respectively. These, along with H⋯O/O⋯H and H⋯C/C⋯H are shown pairwise by type for the two structures in Fig. 6 ▸.

## 4. Database survey

A search of the Cambridge Structural Database (CSD v6.0, April 2025: Groom *et al.*, 2016 ▸) on the common elements of structures **I** and **II** returned six hits, but only two bear any particular similarity to **I** and **II**. CSD refcode XILJIP (Bai *et al.*, 2023 ▸) is a flavone: 7-*tert*-butyl-2-phenyl-4*H*-1-benzo­pyran-4-one (C_19_H_18_O_2_), and ZIYLAU (Kataeva *et al.*, 1995 ▸) is (6*H*)-12-oxo-3-*tert*-butyl-dibenzo[*d*,*g*](1,3)dioxocine, C_18_H_18_O_3_, an eight-membered cyclic acetal. More recently, the crystal structures of etoxazole (C_21_H_23_F_2_NO_2_, CSD deposition 2422554; Sowbhagya *et al.*, 2025*a* ▸) and several metabolites and related compounds have been reported: ‘R4’ (C_21_H_25_F_2_NO_3_, CSD deposition 2487064; Sowbhagya *et al.*, 2025*b* ▸); ‘R13’ (C_21_H_21_F_2_NO_2_, CSD deposition 2397916; Mohan Kumar *et al.*, 2024 ▸); and the bromide and fumarate salts of ‘R7’ (C_21_H_26_BrF_2_NO_3_ and C_25_H_29_F_2_NO_7_, CSD depositions 2533697 and 2533698; Mohan Kumar *et al.*, 2026 ▸).

## 5. Synthesis and crystallization

The samples of compounds **I** and **II** were received as a gift from Honeychem Pharma Research Pvt. Ltd. They were purified by column chromatography and recrystallized from hexane by slow evaporation to obtain colourless crystals.

## 6. Refinement

Crystal data, data collection and structure refinement details are summarized in Table 3 ▸. Carbon-bound hydrogen atoms were found in difference-Fourier maps, but most were subsequently included in the refinement using riding models, with constrained distances set to 0.95 Å (C*sp*^2^—H), 0.98 Å (*R*—CH_3_) and 0.99 Å (*R*_2_—CH_2_). The methyl­ene hydrogen atom H2 in **I** and the methyl hydrogen atoms H11*A*/*B*/*C* were refined in order to obtain standard uncertainties for the C—H⋯π distances (Tables 1 ▸ and 2 ▸). The hydroxyl hydrogen coordinates in **I** were refined. *U*_iso_(H) parameters were set to values of either 1.2*U*_eq_ or 1.5*U*_eq_ (*R—*CH_3_, O–H) of the attached atom. Restraints (*SHELXL* command SADI) were used to ensure satisfactory refinement of methyl hydrogen atoms across the mirror plane of *P*2_1_/*m* in **II**. The numbering schemes start at ‘C2’ (**I** and **II**) and ‘O2’ (**II**) for carbon and oxygen to ensure correspondence with the published structures of etoxazole (Sowbhagya *et al.*, 2025*a* ▸) and its ‘R4’ (Sowbhagya *et al.*, 2025*b* ▸), ‘R7’ (Mohan Kumar *et al.*, 2026 ▸), and ‘R13’ metabolites (Mohan Kumar *et al.*, 2024 ▸).

## Supplementary Material

Crystal structure: contains datablock(s) I, II, global. DOI: 10.1107/S2056989026004329/nx2035sup1.cif

Structure factors: contains datablock(s) I. DOI: 10.1107/S2056989026004329/nx2035Isup2.hkl

Structure factors: contains datablock(s) II. DOI: 10.1107/S2056989026004329/nx2035IIsup3.hkl

Supporting information file. DOI: 10.1107/S2056989026004329/nx2035Isup4.cml

Supporting information file. DOI: 10.1107/S2056989026004329/nx2035IIsup5.cml

CCDC references: 2549023, 2549022

Additional supporting information:  crystallographic information; 3D view; checkCIF report

## Figures and Tables

**Figure 2:**
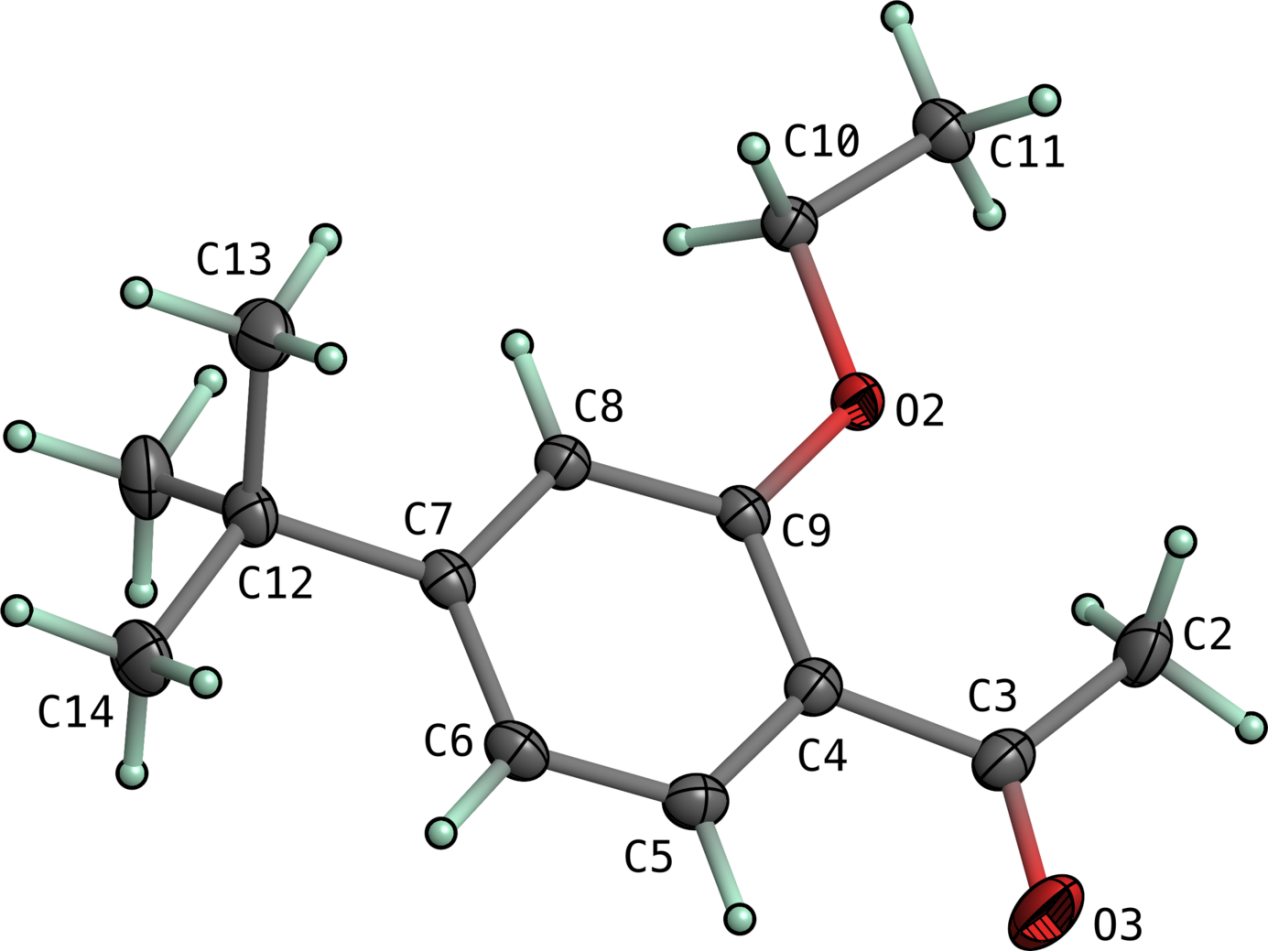
An ellipsoid plot of **II** (50% probability). Hydrogen atoms are drawn as small arbitrary spheres.

**Figure 5:**
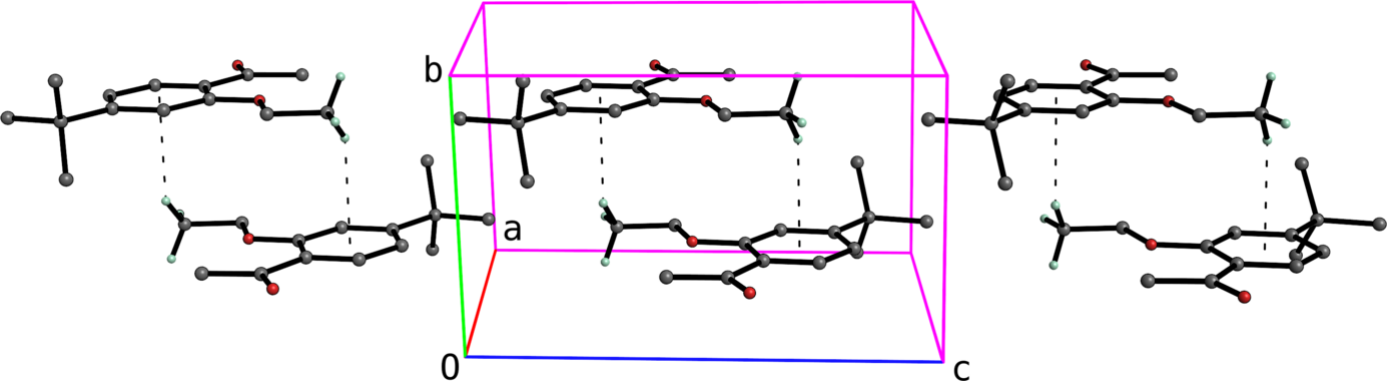
A perspective partial packing plot of **II** viewed slightly off the *a* axis. C—H⋯π contacts are drawn as thin dashed lines, which connect the mol­ecules into columns that extend parallel to the *b* axis.

**Table 1:** Hydrogen bonds and close contacts (Å, °) in **I**

*D*—H⋯*A*	*D*—H	H⋯*A*	*D*⋯*A*	*D*—H⋯*A*
O1—H1*O*⋯O3	0.88 (2)	1.94 (2)	2.5518 (16)	125.1 (18)
C11—H11*A*⋯O1^i^	0.98	2.60	3.3885 (19)	137.2
				
C—H⋯centroid				
C2—H2⋯*Cg*_C4–C9_^ii^			2.647 (12)	

Symmetry codes: (i) *x*, *y*, *z* − 1; (ii) 1 − *x*, 1 − *y*, 1 − *z*.

**Table 2:** Close contacts (Å, °) in **II**

*D*—H⋯*A*	*D*—H	H⋯*A*	*D*⋯*A*	*D*—H⋯*A*
C8—H8⋯O3^i^	0.95	2.51	3.4535 (15)	175.5
				
C—H⋯centroid				
C11—H11*B*⋯*Cg*_C4–C9_^ii^			2.736 (17)	

Symmetry codes: (i) *x* − 1, *y*, *z*; (ii) 1 − *x*, 1 − *y*, 1 − *z*.

**Table 3:** Experimental details

	**I**	**II**
Crystal data		
Chemical formula	C_14_H_20_O_3_	C_14_H_20_O_2_
*M*_r_	236.30	220.30
Crystal system, space group	Orthorhombic, *Pnma*	Monoclinic, *P*2_1_/*m*
Temperature (K)	100	100
*a*, *b*, *c* (Å)	22.0186 (7), 6.8796 (3), 8.4858 (3)	8.0183 (3), 7.0193 (2), 11.5258 (4)
α, β, γ (°)	90, 90, 90	90, 94.469 (1), 90
*V* (Å^3^)	1285.42 (8)	646.73 (4)
*Z*	4	2
Radiation type	Mo *K*α	Mo *K*α
μ (mm^−1^)	0.08	0.07
Crystal size (mm)	0.27 × 0.20 × 0.19	0.28 × 0.27 × 0.20

Data collection		
Diffractometer	Bruker D8 Venture dual source	Bruker D8 Venture dual source
Absorption correction	Multi-scan (*SADABS*; Krause *et al.*, 2015 ▸)	Multi-scan (*SADABS*;Krause *et al.*, 2015 ▸)
*T*_min_, *T*_max_	0.883, 0.971	0.884, 0.971
No. of measured, independent and observed [*I* > 2σ(*I*)] reflections	18796, 1592, 1391	12368, 1596, 1387
*R*_int_	0.028	0.022
(sin θ/λ)_max_ (Å^−1^)	0.650	0.649

Refinement		
*R*[*F*^2^ > 2σ(*F*^2^)], *wR*(*F*^2^), *S*	0.032, 0.072, 1.12	0.031, 0.077, 1.06
No. of reflections	1592	1596
No. of parameters	107	111
No. of restraints	0	18
H-atom treatment	H atoms treated by a mixture of independent and constrained refinement	H atoms treated by a mixture of independent and constrained refinement
Δρ_max_, Δρ_min_ (e Å^−3^)	0.29, −0.18	0.24, −0.18

Computer programs: *APEX5* (Bruker, 2023 ▸), *SHELXT* (Sheldrick, 2015*a* ▸), *SHELXL2025/1* (Sheldrick, 2015*b* ▸), *PrimeXP* (Parkin, 2026 ▸), *Overlay* (Parkin, 2025 ▸), *SHELX* (Sheldrick, 2008 ▸) and *publCIF* (Westrip, 2010 ▸).

## supplementary crystallographic information

### 1-(4-tert-Butyl-2-ethoxyphenyl)-2-hydroxyethan-1-one (I) Crystal data

**Table:**

C_14_H_20_O_3_	*D*_x_ = 1.221 Mg m^−^^3^
*M**_r_* = 236.30	Mo *K*α radiation, λ = 0.71073 Å
Orthorhombic, *P**n**m**a*	Cell parameters from 9900 reflections
*a* = 22.0186 (7) Å	θ = 2.6–27.5°
*b* = 6.8796 (3) Å	µ = 0.08 mm^−^^1^
*c* = 8.4858 (3) Å	*T* = 100 K
*V* = 1285.42 (8) Å^3^	Cut block, colourless
*Z* = 4	0.27 × 0.20 × 0.19 mm
*F*(000) = 512	

### 1-(4-tert-Butyl-2-ethoxyphenyl)-2-hydroxyethan-1-one (I) Data collection

**Table:**

Bruker D8 Venture dual source diffractometer	1592 independent reflections
Radiation source: microsource	1391 reflections with *I* > 2σ(*I*)
Detector resolution: 7.41 pixels mm^-1^	*R*_int_ = 0.028
φ and ω scans	θ_max_ = 27.5°, θ_min_ = 2.6°
Absorption correction: multi-scan (*SADABS*; Krause *et al*., 2015)	*h* = −28→26
*T*_min_ = 0.883, *T*_max_ = 0.971	*k* = −8→8
18796 measured reflections	*l* = −11→11

### 1-(4-tert-Butyl-2-ethoxyphenyl)-2-hydroxyethan-1-one (I) Refinement

**Table:**

Refinement on *F*^2^	Secondary atom site location: difference Fourier map
Least-squares matrix: full	Hydrogen site location: mixed
*R*[*F*^2^ > 2σ(*F*^2^)] = 0.032	H atoms treated by a mixture of independent and constrained refinement
*wR*(*F*^2^) = 0.072	*w* = 1/[σ^2^(*F*_o_^2^) + (0.0104*P*)^2^ + 0.6298*P*] where *P* = (*F*_o_^2^ + 2*F*_c_^2^)/3
*S* = 1.12	(Δ/σ)_max_ < 0.001
1592 reflections	Δρ_max_ = 0.29 e Å^−^^3^
107 parameters	Δρ_min_ = −0.18 e Å^−^^3^
0 restraints	Extinction correction: SHELXL-2025/1 (Sheldrick 2015b), Fc^*^=kFc[1+0.001xFc^2^λ^3^/sin(2θ)]^-1/4^
Primary atom site location: structure-invariant direct methods	Extinction coefficient: 0.0042 (10)

### 1-(4-tert-Butyl-2-ethoxyphenyl)-2-hydroxyethan-1-one (I) Special details

**Table:**

Experimental. The crystal was mounted using polyisobutene oil on the tip of a fine glass fibre, which was fastened in a copper mounting pin with electrical solder. It was placed directly into the cold gas stream of a liquid-nitrogen based cryostat (Hope, 1994; Parkin & Hope, 1998). Diffraction data were collected with the crystal at 100K.
Geometry. All esds (except the esd in the dihedral angle between two l.s. planes) are estimated using the full covariance matrix. The cell esds are taken into account individually in the estimation of esds in distances, angles and torsion angles; correlations between esds in cell parameters are only used when they are defined by crystal symmetry. An approximate (isotropic) treatment of cell esds is used for estimating esds involving l.s. planes.
Refinement. Refinement progress was checked using *Platon* (Spek, 2020) and by an *R*-tensor (Parkin, 2000). The final model was further checked with the IUCr utility *checkCIF*.

### 1-(4-tert-Butyl-2-ethoxyphenyl)-2-hydroxyethan-1-one (I) Fractional atomic coordinates and isotropic or equivalent isotropic displacement parameters (Å^2^)

**Table:**

	*x*	*y*	*z*	*U*_iso_*/*U*_eq_	Occ. (<1)
O1	0.40136 (5)	0.250000	0.80021 (13)	0.0255 (3)	
H1O	0.4318 (9)	0.250000	0.867 (2)	0.038*	
O2	0.46532 (4)	0.250000	0.34710 (11)	0.0189 (2)	
O3	0.51720 (5)	0.250000	0.80930 (12)	0.0280 (3)	
C5	0.60380 (7)	0.250000	0.57638 (17)	0.0193 (3)	
H5	0.615895	0.250000	0.683843	0.023*	
C6	0.64763 (7)	0.250000	0.46117 (17)	0.0190 (3)	
H6	0.689280	0.250000	0.490378	0.023*	
C7	0.63182 (6)	0.250000	0.30110 (17)	0.0168 (3)	
C8	0.57029 (6)	0.250000	0.26240 (17)	0.0164 (3)	
H8	0.558521	0.250000	0.154695	0.020*	
C9	0.52563 (6)	0.250000	0.37944 (17)	0.0156 (3)	
C10	0.44505 (6)	0.250000	0.18579 (16)	0.0183 (3)	
H10A	0.459919	0.133001	0.129884	0.022*	0.5
H10B	0.459919	0.366999	0.129884	0.022*	0.5
C11	0.37648 (7)	0.250000	0.19426 (18)	0.0226 (3)	
H11A	0.359668	0.250000	0.087337	0.034*	
H11B	0.362664	0.133690	0.250476	0.034*	0.5
H11C	0.362664	0.366310	0.250476	0.034*	0.5
C12	0.68190 (6)	0.250000	0.17624 (17)	0.0182 (3)	
C13	0.65627 (7)	0.250000	0.00871 (17)	0.0237 (3)	
H13A	0.689793	0.250000	−0.067248	0.036*	
H13B	0.631291	0.133690	−0.006987	0.036*	0.5
H13C	0.631291	0.366310	−0.006987	0.036*	0.5
C14	0.72128 (5)	0.43293 (17)	0.19745 (13)	0.0241 (2)	
H14A	0.753264	0.434623	0.117157	0.036*	
H14B	0.739798	0.431616	0.302466	0.036*	
H14C	0.695857	0.549033	0.186255	0.036*	
C2	0.43015 (7)	0.250000	0.65125 (17)	0.0187 (3)	
H2	0.4181 (5)	0.3657 (17)	0.5896 (13)	0.022*	
C3	0.49835 (7)	0.250000	0.67329 (17)	0.0186 (3)	
C4	0.54169 (6)	0.250000	0.53968 (16)	0.0166 (3)	

### 1-(4-tert-Butyl-2-ethoxyphenyl)-2-hydroxyethan-1-one (I) Atomic displacement parameters (Å^2^)

**Table:**

	*U*^11^	*U*^22^	*U*^33^	*U*^12^	*U*^13^	*U*^23^
O1	0.0260 (6)	0.0333 (6)	0.0172 (5)	0.000	0.0060 (5)	0.000
O2	0.0146 (5)	0.0291 (6)	0.0131 (5)	0.000	−0.0009 (4)	0.000
O3	0.0278 (6)	0.0418 (7)	0.0146 (5)	0.000	0.0000 (4)	0.000
C5	0.0235 (7)	0.0191 (7)	0.0155 (7)	0.000	−0.0036 (6)	0.000
C6	0.0171 (7)	0.0191 (7)	0.0209 (7)	0.000	−0.0038 (6)	0.000
C7	0.0182 (7)	0.0136 (7)	0.0186 (7)	0.000	0.0005 (6)	0.000
C8	0.0185 (7)	0.0162 (7)	0.0145 (6)	0.000	−0.0008 (5)	0.000
C9	0.0156 (7)	0.0137 (6)	0.0177 (7)	0.000	−0.0016 (5)	0.000
C10	0.0185 (7)	0.0245 (8)	0.0118 (6)	0.000	−0.0016 (5)	0.000
C11	0.0181 (7)	0.0301 (8)	0.0195 (7)	0.000	−0.0017 (6)	0.000
C12	0.0158 (7)	0.0198 (7)	0.0191 (7)	0.000	0.0006 (5)	0.000
C13	0.0191 (7)	0.0333 (9)	0.0188 (7)	0.000	0.0027 (6)	0.000
C14	0.0207 (5)	0.0246 (6)	0.0270 (5)	−0.0029 (4)	0.0020 (4)	0.0007 (5)
C2	0.0223 (7)	0.0190 (7)	0.0147 (7)	0.000	0.0039 (6)	0.000
C3	0.0248 (7)	0.0143 (7)	0.0165 (7)	0.000	−0.0004 (6)	0.000
C4	0.0197 (7)	0.0144 (7)	0.0159 (7)	0.000	−0.0001 (6)	0.000

### 1-(4-tert-Butyl-2-ethoxyphenyl)-2-hydroxyethan-1-one (I) Geometric parameters (Å, º)

**Table:**

O1—C2	1.4141 (17)	C10—H10B	0.9900
O1—H1O	0.88 (2)	C11—H11A	0.9800
O2—C9	1.3562 (16)	C11—H11B	0.9800
O2—C10	1.4397 (16)	C11—H11C	0.9800
O3—C3	1.2265 (18)	C12—C13	1.530 (2)
C5—C6	1.374 (2)	C12—C14	1.5389 (13)
C5—C4	1.403 (2)	C12—C14^i^	1.5389 (13)
C5—H5	0.9500	C13—H13A	0.9800
C6—C7	1.402 (2)	C13—H13B	0.9800
C6—H6	0.9500	C13—H13C	0.9800
C7—C8	1.394 (2)	C14—H14A	0.9800
C7—C12	1.529 (2)	C14—H14B	0.9800
C8—C9	1.398 (2)	C14—H14C	0.9800
C8—H8	0.9500	C2—C3	1.513 (2)
C9—C4	1.4049 (19)	C2—H2	0.989 (12)
C10—C11	1.511 (2)	C3—C4	1.482 (2)
C10—H10A	0.9900		
			
C2—O1—H1O	103.6 (13)	H11B—C11—H11C	109.5
C9—O2—C10	119.73 (11)	C7—C12—C13	112.21 (12)
C6—C5—C4	121.80 (13)	C7—C12—C14	108.98 (8)
C6—C5—H5	119.1	C13—C12—C14	108.46 (8)
C4—C5—H5	119.1	C7—C12—C14^i^	108.98 (8)
C5—C6—C7	121.00 (13)	C13—C12—C14^i^	108.46 (8)
C5—C6—H6	119.5	C14—C12—C14^i^	109.72 (12)
C7—C6—H6	119.5	C12—C13—H13A	109.5
C8—C7—C6	118.00 (13)	C12—C13—H13B	109.5
C8—C7—C12	122.52 (13)	H13A—C13—H13B	109.5
C6—C7—C12	119.48 (13)	C12—C13—H13C	109.5
C7—C8—C9	121.09 (13)	H13A—C13—H13C	109.5
C7—C8—H8	119.5	H13B—C13—H13C	109.5
C9—C8—H8	119.5	C12—C14—H14A	109.5
O2—C9—C8	123.04 (13)	C12—C14—H14B	109.5
O2—C9—C4	116.25 (12)	H14A—C14—H14B	109.5
C8—C9—C4	120.72 (13)	C12—C14—H14C	109.5
O2—C10—C11	105.33 (11)	H14A—C14—H14C	109.5
O2—C10—H10A	110.7	H14B—C14—H14C	109.5
C11—C10—H10A	110.7	O1—C2—C3	109.53 (12)
O2—C10—H10B	110.7	O1—C2—H2	110.7 (7)
C11—C10—H10B	110.7	C3—C2—H2	109.3 (7)
H10A—C10—H10B	108.8	O3—C3—C4	120.14 (14)
C10—C11—H11A	109.5	O3—C3—C2	116.88 (13)
C10—C11—H11B	109.5	C4—C3—C2	122.98 (13)
H11A—C11—H11B	109.5	C5—C4—C9	117.40 (13)
C10—C11—H11C	109.5	C5—C4—C3	117.26 (13)
H11A—C11—H11C	109.5	C9—C4—C3	125.34 (13)
			
C4—C5—C6—C7	0.000 (1)	C8—C7—C12—C14^i^	−120.14 (8)
C5—C6—C7—C8	0.000 (1)	C6—C7—C12—C14^i^	59.86 (8)
C5—C6—C7—C12	180.000 (1)	O1—C2—C3—O3	0.000 (1)
C6—C7—C8—C9	0.000 (1)	O1—C2—C3—C4	180.000 (1)
C12—C7—C8—C9	180.000 (1)	C6—C5—C4—C9	0.000 (1)
C10—O2—C9—C8	0.000 (1)	C6—C5—C4—C3	180.000 (1)
C10—O2—C9—C4	180.000 (1)	O2—C9—C4—C5	180.000 (1)
C7—C8—C9—O2	180.000 (1)	C8—C9—C4—C5	0.000 (1)
C7—C8—C9—C4	0.000 (1)	O2—C9—C4—C3	0.000 (1)
C9—O2—C10—C11	180.0	C8—C9—C4—C3	180.000 (1)
C8—C7—C12—C13	0.000 (1)	O3—C3—C4—C5	0.000 (1)
C6—C7—C12—C13	180.0	C2—C3—C4—C5	180.000 (1)
C8—C7—C12—C14	120.14 (8)	O3—C3—C4—C9	180.000 (1)
C6—C7—C12—C14	−59.86 (8)	C2—C3—C4—C9	0.000 (1)

Symmetry code: (i) *x*, −*y*+1/2, *z*.

### 1-(4-tert-Butyl-2-ethoxyphenyl)-2-hydroxyethan-1-one (I) Hydrogen-bond geometry (Å, º)

**Table:**

*D*—H···*A*	*D*—H	H···*A*	*D*···*A*	*D*—H···*A*
O1—H1*O*···O3	0.88 (2)	1.94 (2)	2.5518 (16)	125 (2)
C11—H11*A*···O1^ii^	0.98	2.60	3.3885 (19)	137

Symmetry code: (ii) *x*, *y*, *z*−1.

### 1-(4-tert-Butyl-2-ethoxyphenyl)ethan-1-one (II) Crystal data

**Table:**

C_14_H_20_O_2_	*F*(000) = 240
*M**_r_* = 220.30	*D*_x_ = 1.131 Mg m^−^^3^
Monoclinic, *P*2_1_/*m*	Mo *K*α radiation, λ = 0.71073 Å
*a* = 8.0183 (3) Å	Cell parameters from 8769 reflections
*b* = 7.0193 (2) Å	θ = 2.6–27.4°
*c* = 11.5258 (4) Å	µ = 0.07 mm^−^^1^
β = 94.469 (1)°	*T* = 100 K
*V* = 646.73 (4) Å^3^	Wedge-shaped block, colourless
*Z* = 2	0.28 × 0.27 × 0.20 mm

### 1-(4-tert-Butyl-2-ethoxyphenyl)ethan-1-one (II) Data collection

**Table:**

Bruker D8 Venture dual source diffractometer	1596 independent reflections
Radiation source: microsource	1387 reflections with *I* > 2σ(*I*)
Detector resolution: 7.41 pixels mm^-1^	*R*_int_ = 0.022
φ and ω scans	θ_max_ = 27.5°, θ_min_ = 2.6°
Absorption correction: multi-scan (*SADABS*;Krause *et al*., 2015)	*h* = −10→10
*T*_min_ = 0.884, *T*_max_ = 0.971	*k* = −8→9
12368 measured reflections	*l* = −14→14

### 1-(4-tert-Butyl-2-ethoxyphenyl)ethan-1-one (II) Refinement

**Table:**

Refinement on *F*^2^	Primary atom site location: structure-invariant direct methods
Least-squares matrix: full	Secondary atom site location: difference Fourier map
*R*[*F*^2^ > 2σ(*F*^2^)] = 0.031	Hydrogen site location: mixed
*wR*(*F*^2^) = 0.077	H atoms treated by a mixture of independent and constrained refinement
*S* = 1.06	*w* = 1/[σ^2^(*F*_o_^2^) + (0.0258*P*)^2^ + 0.178*P*] where *P* = (*F*_o_^2^ + 2*F*_c_^2^)/3
1596 reflections	(Δ/σ)_max_ < 0.001
111 parameters	Δρ_max_ = 0.24 e Å^−^^3^
18 restraints	Δρ_min_ = −0.18 e Å^−^^3^

### 1-(4-tert-Butyl-2-ethoxyphenyl)ethan-1-one (II) Special details

**Table:**

Experimental. The crystal was mounted using polyisobutene oil on the tip of a fine glass fibre, which was fastened in a copper mounting pin with electrical solder. It was placed directly into the cold gas stream of a liquid-nitrogen based cryostat (Hope, 1994; Parkin & Hope, 1998). Diffraction data were collected with the crystal at 100K.
Geometry. All esds (except the esd in the dihedral angle between two l.s. planes) are estimated using the full covariance matrix. The cell esds are taken into account individually in the estimation of esds in distances, angles and torsion angles; correlations between esds in cell parameters are only used when they are defined by crystal symmetry. An approximate (isotropic) treatment of cell esds is used for estimating esds involving l.s. planes.
Refinement. Refinement progress was checked using *Platon* (Spek, 2020) and by an *R*-tensor (Parkin, 2000). The final model was further checked with the IUCr utility *checkCIF*.

### 1-(4-tert-Butyl-2-ethoxyphenyl)ethan-1-one (II) Fractional atomic coordinates and isotropic or equivalent isotropic displacement parameters (Å^2^)

**Table:**

	*x*	*y*	*z*	*U*_iso_*/*U*_eq_	Occ. (<1)
O2	0.54443 (10)	0.750000	0.51826 (7)	0.0200 (2)	
O3	1.01603 (12)	0.750000	0.39884 (9)	0.0445 (3)	
C2	0.88242 (16)	0.750000	0.57222 (12)	0.0304 (3)	
H2A	0.8247 (18)	0.863 (2)	0.5989 (11)	0.046*	0.5
H2B	0.9971 (14)	0.750000	0.6077 (10)	0.046*	
H2C	0.8247 (18)	0.637 (2)	0.5989 (11)	0.046*	0.5
C3	0.88279 (15)	0.750000	0.44258 (11)	0.0233 (3)	
C4	0.72412 (15)	0.750000	0.36485 (10)	0.0193 (3)	
C5	0.74016 (15)	0.750000	0.24516 (11)	0.0220 (3)	
H5	0.849183	0.750000	0.218261	0.026*	
C6	0.60365 (16)	0.750000	0.16426 (10)	0.0216 (3)	
H6	0.620143	0.750000	0.083527	0.026*	
C7	0.44184 (15)	0.750000	0.20052 (10)	0.0181 (2)	
C8	0.42282 (14)	0.750000	0.3200 (1)	0.0177 (2)	
H8	0.313385	0.750000	0.346210	0.021*	
C9	0.56034 (14)	0.750000	0.40159 (10)	0.0168 (2)	
C10	0.37896 (14)	0.750000	0.5582 (1)	0.0184 (2)	
H10A	0.316638	0.635502	0.529387	0.022*	0.5
H10B	0.316638	0.864498	0.529387	0.022*	0.5
C11	0.39854 (16)	0.750000	0.68936 (11)	0.0222 (3)	
H11A	0.2880 (14)	0.750000	0.7205 (9)	0.033*	
H11B	0.4598 (17)	0.637 (2)	0.7182 (10)	0.033*	0.5
H11C	0.4598 (17)	0.863 (2)	0.7182 (10)	0.033*	0.5
C12	0.28480 (16)	0.750000	0.11559 (10)	0.0218 (3)	
C13	0.32689 (18)	0.750000	−0.01188 (11)	0.0299 (3)	
H13A	0.223093	0.750000	−0.062750	0.045*	
H13B	0.392239	0.863995	−0.027190	0.045*	0.5
H13C	0.392239	0.636005	−0.027190	0.045*	0.5
C14	0.18143 (12)	0.57111 (15)	0.13709 (8)	0.0296 (2)	
H14A	0.081042	0.569519	0.082847	0.044*	
H14B	0.148547	0.572309	0.217212	0.044*	
H14C	0.248705	0.457326	0.124919	0.044*	

### 1-(4-tert-Butyl-2-ethoxyphenyl)ethan-1-one (II) Atomic displacement parameters (Å^2^)

**Table:**

	*U*^11^	*U*^22^	*U*^33^	*U*^12^	*U*^13^	*U*^23^
O2	0.0179 (4)	0.0284 (5)	0.0137 (4)	0.000	0.0006 (3)	0.000
O3	0.0184 (5)	0.0828 (9)	0.0325 (6)	0.000	0.0020 (4)	0.000
C2	0.0202 (6)	0.0447 (9)	0.0254 (7)	0.000	−0.0046 (5)	0.000
C3	0.0187 (6)	0.0240 (6)	0.0272 (7)	0.000	0.0012 (5)	0.000
C4	0.0191 (6)	0.0182 (6)	0.0206 (6)	0.000	0.0011 (4)	0.000
C5	0.0204 (6)	0.0231 (6)	0.0231 (6)	0.000	0.0056 (5)	0.000
C6	0.0268 (6)	0.0218 (6)	0.0167 (6)	0.000	0.0048 (5)	0.000
C7	0.0231 (6)	0.0146 (5)	0.0166 (5)	0.000	0.0008 (4)	0.000
C8	0.0182 (5)	0.0177 (6)	0.0173 (5)	0.000	0.0018 (4)	0.000
C9	0.0201 (6)	0.0147 (5)	0.0156 (5)	0.000	0.0012 (4)	0.000
C10	0.0182 (6)	0.0203 (6)	0.0169 (5)	0.000	0.0021 (4)	0.000
C11	0.0269 (6)	0.0235 (6)	0.0164 (6)	0.000	0.0021 (5)	0.000
C12	0.0247 (6)	0.0245 (6)	0.0157 (6)	0.000	−0.0014 (4)	0.000
C13	0.0341 (7)	0.0393 (8)	0.0157 (6)	0.000	−0.0017 (5)	0.000
C14	0.0308 (5)	0.0349 (5)	0.0220 (4)	−0.0084 (4)	−0.0052 (3)	−0.0009 (4)

### 1-(4-tert-Butyl-2-ethoxyphenyl)ethan-1-one (II) Geometric parameters (Å, º)

**Table:**

O2—C9	1.3607 (14)	C8—H8	0.9500
O2—C10	1.4379 (14)	C10—C11	1.5076 (16)
O3—C3	1.2163 (15)	C10—H10A	0.9900
C2—C3	1.4945 (18)	C10—H10B	0.9900
C2—H2A	0.979 (11)	C11—H11A	0.982 (10)
C2—H2B	0.977 (11)	C11—H11B	0.978 (10)
C2—H2C	0.979 (11)	C11—H11C	0.978 (10)
C3—C4	1.4978 (16)	C12—C13	1.5329 (17)
C4—C5	1.3955 (17)	C12—C14^i^	1.5353 (12)
C4—C9	1.4109 (16)	C12—C14	1.5353 (12)
C5—C6	1.3815 (17)	C13—H13A	0.9800
C5—H5	0.9500	C13—H13B	0.9800
C6—C7	1.3937 (17)	C13—H13C	0.9800
C6—H6	0.9500	C14—H14A	0.9800
C7—C8	1.3973 (16)	C14—H14B	0.9800
C7—C12	1.5335 (16)	C14—H14C	0.9800
C8—C9	1.3923 (16)		
			
C9—O2—C10	118.45 (9)	O2—C10—H10A	110.3
C3—C2—H2A	110.6 (8)	C11—C10—H10A	110.3
C3—C2—H2B	110.1 (8)	O2—C10—H10B	110.3
H2A—C2—H2B	108.7 (10)	C11—C10—H10B	110.3
C3—C2—H2C	110.6 (8)	H10A—C10—H10B	108.5
H2A—C2—H2C	108.0 (9)	C10—C11—H11A	109.9 (7)
H2B—C2—H2C	108.7 (10)	C10—C11—H11B	110.5 (8)
O3—C3—C2	118.99 (11)	H11A—C11—H11B	108.6 (9)
O3—C3—C4	118.99 (12)	C10—C11—H11C	110.5 (8)
C2—C3—C4	122.02 (11)	H11A—C11—H11C	108.6 (9)
C5—C4—C9	117.15 (11)	H11B—C11—H11C	108.6 (8)
C5—C4—C3	116.87 (11)	C13—C12—C7	112.38 (10)
C9—C4—C3	125.99 (11)	C13—C12—C14^i^	108.46 (7)
C6—C5—C4	122.55 (11)	C7—C12—C14^i^	108.89 (6)
C6—C5—H5	118.7	C13—C12—C14	108.46 (7)
C4—C5—H5	118.7	C7—C12—C14	108.89 (6)
C5—C6—C7	120.32 (11)	C14^i^—C12—C14	109.74 (11)
C5—C6—H6	119.8	C12—C13—H13A	109.5
C7—C6—H6	119.8	C12—C13—H13B	109.5
C6—C7—C8	118.11 (11)	H13A—C13—H13B	109.5
C6—C7—C12	123.08 (10)	C12—C13—H13C	109.5
C8—C7—C12	118.81 (10)	H13A—C13—H13C	109.5
C9—C8—C7	121.61 (11)	H13B—C13—H13C	109.5
C9—C8—H8	119.2	C12—C14—H14A	109.5
C7—C8—H8	119.2	C12—C14—H14B	109.5
O2—C9—C8	122.49 (10)	H14A—C14—H14B	109.5
O2—C9—C4	117.24 (10)	C12—C14—H14C	109.5
C8—C9—C4	120.27 (10)	H14A—C14—H14C	109.5
O2—C10—C11	107.12 (9)	H14B—C14—H14C	109.5
			
O3—C3—C4—C5	0.000 (1)	C7—C8—C9—O2	180.000 (1)
C2—C3—C4—C5	180.000 (1)	C7—C8—C9—C4	0.000 (1)
O3—C3—C4—C9	180.000 (1)	C5—C4—C9—O2	180.000 (1)
C2—C3—C4—C9	0.000 (1)	C3—C4—C9—O2	0.000 (1)
C9—C4—C5—C6	0.000 (1)	C5—C4—C9—C8	0.000 (1)
C3—C4—C5—C6	180.000 (1)	C3—C4—C9—C8	180.000 (1)
C4—C5—C6—C7	0.000 (1)	C9—O2—C10—C11	180.000 (1)
C5—C6—C7—C8	0.000 (1)	C6—C7—C12—C13	0.000 (1)
C5—C6—C7—C12	180.000 (1)	C8—C7—C12—C13	180.000 (1)
C6—C7—C8—C9	0.000 (1)	C6—C7—C12—C14^i^	−120.18 (7)
C12—C7—C8—C9	180.000 (1)	C8—C7—C12—C14^i^	59.82 (7)
C10—O2—C9—C8	0.000 (1)	C6—C7—C12—C14	120.18 (7)
C10—O2—C9—C4	180.000 (1)	C8—C7—C12—C14	−59.82 (7)

Symmetry code: (i) *x*, −*y*+3/2, *z*.

### 1-(4-tert-Butyl-2-ethoxyphenyl)ethan-1-one (II) Hydrogen-bond geometry (Å, º)

**Table:**

*D*—H···*A*	*D*—H	H···*A*	*D*···*A*	*D*—H···*A*
C8—H8···O3^ii^	0.95	2.51	3.4535 (15)	176

Symmetry code: (ii) *x*−1, *y*, *z*.
